# Which anthropometric measurements including visceral fat, subcutaneous fat, body mass index, and waist circumference could predict the urinary stone composition most?

**DOI:** 10.1186/s12894-015-0013-x

**Published:** 2015-03-14

**Authors:** Jae Heon Kim, Seung Whan Doo, Kang Su Cho, Won Jae Yang, Yun Seob Song, Jiyoung Hwang, Seong Sook Hong, Soon-Sun Kwon

**Affiliations:** Department of Urology, Soonchunhyang University Seoul Hospital, Soonchunhyang University College of Medicine, Seoul, Korea; Department of Urology, Gangnam Severance Hospital, Urological Science Institute, Yonsei University College of Medicine, Seoul, Korea; Department of Radiology, Soonchunhyang University Seoul Hospital, Soonchunhyang University College of Medicine Seoul, Seoul, Korea; Biomedical Research Institute, Seoul National University Bundang Hospital, Seongnam, Korea

**Keywords:** Urinary calculi, Obesity, Body mass index, Visceral fat, Computed tomography

## Abstract

**Background:**

Although there is growing evidence of relationship between obesity and some specific stone compositions, results were inconsistent. Due to a greater relationship between metabolic syndrome and some specific stone type, obesity measured by body mass index (BMI) has limitation in determining relationship between obesity and stone compositions. The aim of this study was to determine the relationship among BMI, visceral fat, and stone compositions.

**Methods:**

We retrospectively reviewed data of patients with urinary stone removed over a 5 year period (2011–2014). Data on patient age, gender, BMI, urinary pH, stone composition, fat volumes (including visceral fat, subcutaneous fat, total fat, waist circumference), and ratio for visceral to total fat using computed tomography based delineation were collected. To figure out the predicting factor while adjusting other confounding factors, discriminant analysis was used.

**Results:**

Among 262 cases, average age was 52.21 years. Average BMI and visceral fat were 25.03 cm^2^ and 124.75 cm^2^, respectively. By chi square test, there was significant (*p* < 0.001) difference in stone types according to sex. By ANOVA test, BMI, visceral fat, visceral to subcutaneous fat ratio, the percentage of visceral fat and total fat showed significant association with stone types. By discriminant analysis, visceral fat was proved to be a powerful factor to predict stone composition (structure matrix of visceral fat = −0.735) with 42.0% of predictive value.

**Conclusion:**

Visceral fat adiposity strongly related with uric acid stone and has better predictive value than BMI or urinary pH to classify the types of stone.

## Background

The etiology of urinary stone disease is multifactorial [1]. The prevalence and incidence of urinary stone disease have been reported to be associated with body weight and body mass index (BMI) [2,3]. Obesity is associated with changes in chemical components of serum and urine such as citrate, phosphate, oxalate, and uric acid, resulting in frequent formation of uric acid and calcium oxalate stone [4,5]. Although there is growing evidence of relation between obesity and some specific stone composition, such studies have retrospective nature which suffers from the lack of data and selected cohort problem. Recent study showed that obesity had little effect on stone composition unless the BMI was over 40 [6], highlighting the limitation of recent studies that failed to considering visceral fat adiposity, the main chemical change in urine that is correlated with metabolic syndrome.

To overcome such limitation, we performed a study to investigate the relationship between stone compositions and obesity including visceral obesity. To measure visceral fat adiposity together with subcutaneous and total fat, we reanalyzed computed tomography (CT) using fat measurement program [7]. Our previous efforts in finding the association between stone composition and visceral fat measured by CT delineation method showed meaningful results [8]. However, diverse anthropometric measurement including visceral fat, total fat, visceral fat ratio, and waist circumferences were not determined. No effort has been made to find a predictive model for stone composition which is pivotal to judge the usefulness of measurement of visceral fat.

Although retrospective study is inevitable for investigating stone compositions, our study highlights the relationship between stone compositions and obesity including visceral fat adiposity. This study adopted a discriminant analysis to adjust age and sexual differences and find the best predicting factor for stone compositions which could be a key factor for preventing stone recurrence.

## Methods

### Data collection

Between January 2011 and December 2012, 283 adult patients underwent surgical intervention (ureteroscopy, percutaneous nephrolithotomy, laparoscopic ureterolithotomy) at Soonchunhyang University hospitals located in Seoul. Of which 265 patients with urinary stones (ureteral or renal) were reviewed in this study after obtaining study approval from the Institutional Review Board of Soonchunhyang University Hospital. Waiver of informed consent was permitted by the Institutional Review Board of Soonchunhyang University Hospital. For those two patients with CT images, informed consents were received. Inclusion criteria were: patients with urinary stone who were available for analysis of stone composition and CT-based fat delineation. Patients’ data including age, gender, BMI, first urinary pH before surgical intervention, and urinary stone composition were recorded in a retrospective database. After acquisition of stone analysis data, 18 cases were excluded due to obscure stone compositions. Finally, a total of 265 cases were included for investigation. Parameters from CT-based delineation, visceral, subcutaneous, total fat, waist circumference, and visceral fat to total fat ratio (V/T) were recorded.

### Definition of obesity

BMI was calculated by dividing the weight (kilograms) by the square of the height (meters). Individual BMI values were stratified into two categories (obese ≥, non-obese < 25 kg/m2) developed for Asia-Oceanian populations of South Korea [9]. Obesity was defined as BMI ≥ 25 and after CT-based fat delineation. Visceral obesity was defined as a visceral fat ≥ 100 cm^2^ [10].

### Stone analysis and definition of composition

The composition of collected stones was analyzed by spectroscopy. Each stone sample was washed and dried. A small portion (1 mg) of each stone sample was mixed with potassium bromide (200 mg KBr), which was powdered and then pressed into small tablet. The tablet was then analyzed by spectroscopy. We classified the specimens as calcium oxalate (CO) stones, mixed CO and calcium oxalate phosphate (COP) stones, or calcium phosphate (CP) stones according to results of the analysis, which indicated the presence of calcium, oxalate, or phosphate regardless of mixed uric acid (UA) components. If the results revealed the presence of UA components only, or UA mixed with calcium components only, those stones were classified as UA stones.

### CT-based fat delineation

CT scan were performed with all subjects in supine position using 64-slice MDCT scanner (Sensation 64, Siemens Medical Solutions, Forchheim, Germany) with 16 mm × 0.75 mm collimation, rotation time 420 ms, and tube voltage at 120 kV.

From each patient, one slice of CT data was collected at the location of umbilicus between fourth and fifth lumbar vertebrae. For CT-volume data, six additional CT images were collected from above and below the umbilicus of each patient, respectively. Visceral adipose tissue of PCF and TAT was quantified by MDCT using a dedicated Aquarius 3D Workstation (TeraRecon, San Mateo, CA, USA). The semi-automatic segmentation technique was developed for the quantification of fat volumes. We traced the region of interest manually and defined fat tissue as pixels within a window of −195 to −45 HU and a window centre of −120 HU.

**Figure 1:**
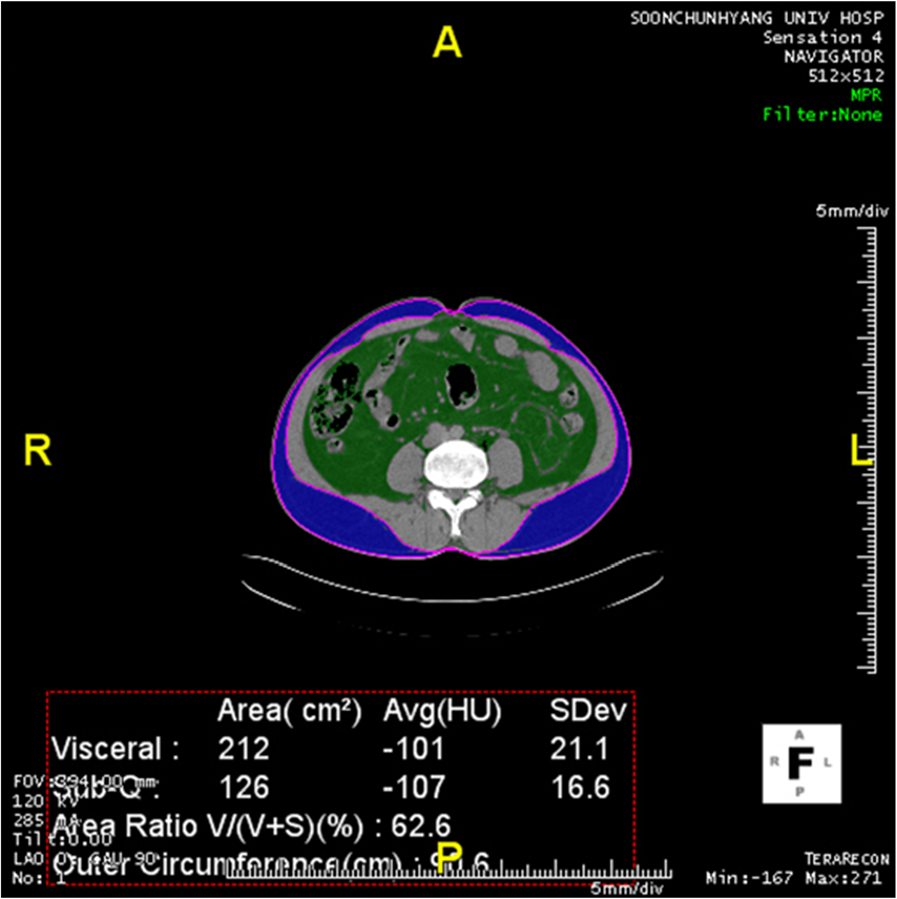
**CT fat delineation in a male with visceral fat obesity without obesity whose visceral fat was 212 cm** ^**2**^ **and BMI was 24.2.** Green color indicates visceral fat, blue color indicates subcutaneous fat.

**Figure 2:**
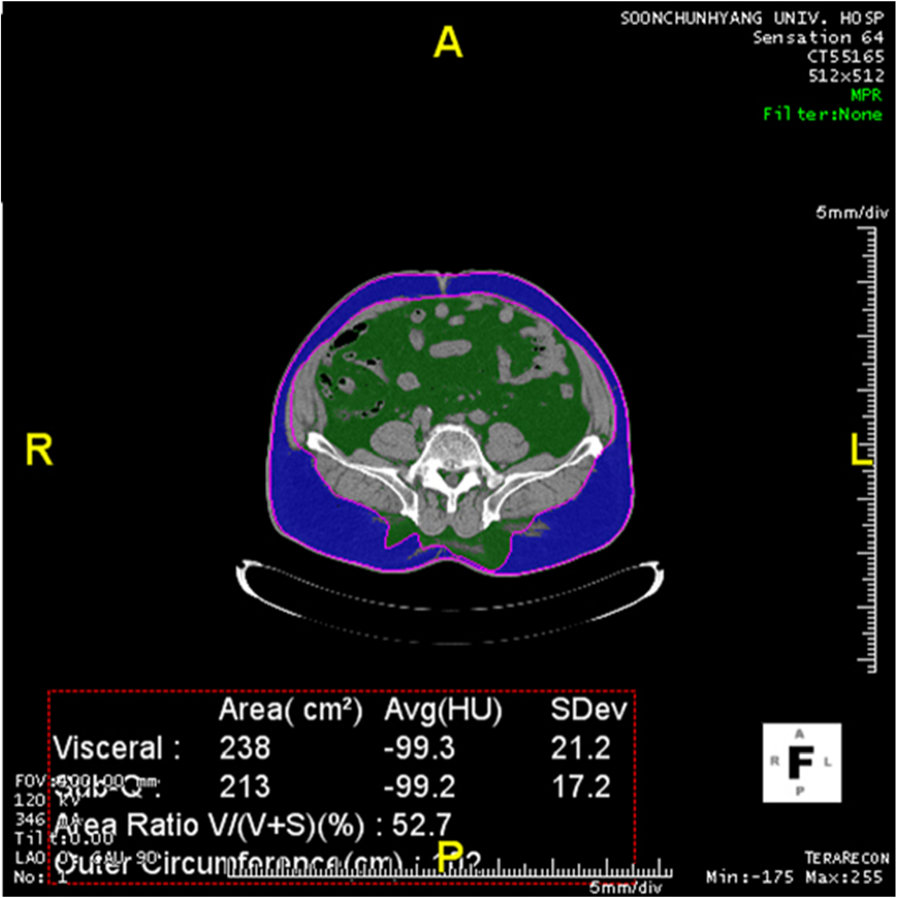
**CT fat delineation in a male with obesity and visceral obesity whose visceral fat was 238 cm** ^**2**^ **and BMI was 30.4.** Green color indicates visceral fat, blue color indicates subcutaneous fat.

Considering the background noise of CT images, all regions below −900 HU were subtracted to remove air compartments. Unnecessary areas such as bed and sheets were removed by labeling all regions followed by removing all regions except the largest region, the body. With all background removed, the segmentation and assessment of body fat were performed only in the area. A threshold binary image showing visceral fat surrounded by subcutaneous fat with bones and other organs was removed by threshold process. The segmentation mask of non-subcutaneous fat area was made to separate regions of visceral fat and subcutaneous fat using color mapping segmentation (Figures 1 and 2).

### Statistical analyses

Mean, standard deviation, and proportion were described. Differences of continuous variables and categorical variables were analyzed by ANOVA and Fisher exact test, respectively. Correlation relationship of continuous variables was analyzed by the Pearson’s correlation coefficient test. Linear discriminant analysis was performed for two or more groups. The goal of the analysis was to classify cases into urinary stone components based on their values for a set of predictor variable. In the analysis, a classification rule was developed using cases for which group membership was known. In the classification, a rule was used to classify cases for which group membership was not known. Grouping categorical and independent variables must be interval or dichotomous since they will be used in a regression-type equation. All statistical analyses were performed using SPSS Statistics Version 20.0 (IBM Corp. USA). All statistics were two-tailed. Statistical significance was considered when *p* value was less than 0.05.

## Results

### Baseline characteristics

The 265 cases included 156 males and 109 females with average age of 52.21 years. Average BMI and visceral fat were 25.03 cm^2^ and 124.75 cm^2^, respectively. By chi-square test, there was significant (*p* < 0.001) difference of stone types according to sex (Table 1). By ANOVA test, stone composition showed significant differences according to visceral fat (*p* < 0.001), visceral to subcutaneous fat ratio (*p* = 0.013), percentage of visceral fat (*p* < 0.001), and total fat (*p* = 0.002), but not significant according to BMI (*p* = 0.21). Subcutaneous fat, waist circumference, and urine pH were not associated with stone composition (*p* = 0.419, 0.080, and 0.071, respectively) (Table 1).

**Table 1:** **Differences according to the type of stone (n = 265)**

**Parameters**	**CO**	**COP**	**CP**	**UA**	**P value**
Age	44.02 ± 6.32	41.24 ± 7.13	39.45 ± 4.34	43.13 ± 8.22	0.231
Male sex(% within sex, % within stone type)	43.6% (62.4%)	19.9% (44.9%)	17.3% (52.9%)	19.2% (83.3%)	0.001^†^
BMI	25.48 ± 4.13	24.85 ± 3.84	23.88 ± 3.88	26.01 ± 2.59	0.21^‡^
Visceral fat (cm^2^)	135.84 ± 58.98	112.84 ± 52.11	94.10 ± 48.31	174.30 ± 52.73	<0.001^‡^
Subcutaneous fat (cm^2^)	158.85 ± 75.93	166.14 ± 73.60	141.89 ± 91.20	156.86 ± 46.88	0.419^‡^
Visceral fat/subcutaneous fat	0.96 ± 0.47	0.83 ± 0.79	0.94 ± 0.80	1.16 ± 0.38	0.013^‡^
Ratio of visceral fat to total fat (%)	46.51 ± 11.48	40.74 ± 13.22	42.43 ± 16.50	52.38 ± 8.36	<0.001^‡^
Outer circumference (cm)	86.51 ± 9.28	83.97 ± 9.77	82.40 ± 10.06	89.76 ± 7.17	0.080^‡^
Total fat (cm^2^)	294.69 ± 112.14	278.98 ± 109.38	235.99 ± 118.08	331.16 ± 85.97	0.002^‡^
Urine pH	5.81 ± 0.89	5.93 ± 1.03	6.14 ± 0.93	5.58 ± 1.06	0.071^‡^

Data are mean ± standard deviation or number. CO: calcium oxalate stone; COP: calcium oxalate phosphate; CP: calcium phosphate; UA: uric acid.

Statistical analysis by ^†^chi-square test or ^‡^one way ANOVA test.

### Correlation analyses among BMI, parameters of CT-based fat delineation, and urinary pH

BMI showed positive correlation with visceral fat (correlation coefficient cc = 0.607, *p* < 0.001), subcutaneous fat (cc = 0.758, *p* < 0.001), waist circumference and total fat (cc = 0.752, *p* < 0.001). BMI had negative correlation with urinary pH (cc = −0.209, *p* = 0.001) (Table 2). Age was correlated with visceral fat (cc = 0.331, *p* < 0.001), visceral to subcutaneous fat ratio (cc = 0.250, *p* < 0.001), the percentage of visceral fat (cc = 0.317, *p* < 0.001), and total fat (cc = 0.153, *p* = 0.013). However, age was not correlated with BMI (cc = 0.031, *p* = 0.616), subcutaneous fat (cc = −0.021, *p* = 0.735), or urine pH (cc = 0.068, *p* = 0.271) (Table 2).

**Table 2:** **Correlation analysis among anthropometric parameters**

**Coefficient**		**Age**	**BMI**	**Visceral fact (cm2)**	**Subcutaneous fat (cm2)**	**Visceral/subcutaneous**	**Ratio V/total (%)**	**Outer circumference (cm)**	**Total fat (cm2)**	**Urine pH**
**P value**										
Age	Coefficient	1	0.031	0.331**	−0.021	0.250**	0.317**	0.068	0.153*	0.117
	P value		0.616	<0.001	0.735	<0.001	<0.001	0.271	0.013	0.057
BMI	Coefficient		1	0.607**	0.758**	−0.096	−0.042	0.752**	0.804**	−0.209**
	P value			<0.001	<0.001	0.123	0.501	<0.001	<0.001	0.001
Visceral fact (cm2)	Coefficient			1	0.391**	0.400**	0.531**	0.706**	0.791**	−0.178**
	P value				<0.001	<0.001	<0.001	<0.001	<0.001	0.004
Subcutaneous fat (cm2)	Coefficient				1	−0.469**	−0.478**	0.699**	0.873**	−0.143*
	P value					<0.001	<0.001	<0.001	<0.001	0.020
Visceral/subcutaneous	Coefficient					1	0.898**	−0.018	−0.100	−0.076
	P value						<0.001	0.778	0.108	0.222
Ratio V/total (%)	Coefficient						1	0.050	−0.036	−0.083
	P value							0.419	0.557	0.181
Waist circumference (cm)	Coefficient							1	0.839**	−0.238**
	P value								<0.001	<0.001
Total fat (cm2)	Coefficient								1	−0.183**
	P value									0.003
Urine pH	Coefficient									1
	P value									

Ratio V/Total: Ratio of visceral fat to total fat.

*Correlation is significant at the 0.05 level (2-tailed).

**Correlation is significant at the 0.01 level (2-tailed).

### Predictive value for stone composition

By discriminant analysis, canonical correlation was 0.520 with cumulative 85.4%. Wilk’s lambda value was 0.85 (p-value < 0.001). Visceral fat was proved to be the most powerful predictive factor in the structure matrix (−0.735), which was the largest absolute correlation between each variable and any discriminant function (Table 3). By classification results with predictive factor of visceral fat, 42.0% of original grouped cases were correctly classified (Table 4).

**Table 3:** **Canonical correlation and structure matrix function for discriminating the types of stone compositions**

	**Structure matrix function**		
	**1**	**2**	**3**
Viceral fact (cm2)	-.735*	-.011	.157
Outer circumference (cm)	-.411*	-.149	.210
Total fat (cm2)	-.398*	-.242	-.243
Age	-.267*	.023	.025
urine pH	.368	-.581*	-.276
Sex	.251	.313*	.091
Ratio of visceral fat to total fat (%)	-.278	.094	.730*
	-.457	.385	.594*
	-.046	-.347	-.471*
BMI	-.299	-.001	-.466*
	**Canonical discriminant function**		
	**1**	**2**	**3**
Sex	.733	-.605	-.035
Age	-.023	-.014	.005
BMI	-.069	.091	-.244
Viceral fact (cm2)	-.030	-.015	-.001
Subcutaneous fat (cm2)	.011	-.002	-.001
Visceral/subcutaneous	.426	−2.099	1.646
Ratio of visceral fat to total fat (%)	.062	.147	-.037
Outer circumference (cm)	.057	.044	.082
urine pH	.278	.436	.117
(Constant)	−5.767	−9.336	−1.388

Pooled within-groups correlations between discriminating variables and standardized canonical discriminant functions.

*Largest absolute correlation between each variable and any discriminant function.

**Table 4:** **Classification analysis of predicted grouping of stone type**

		**Stone type**	**Predicted group membership**				**Total**
			**CO**	**COP**	**CP**	**UA**	
Original	Count	CO	30	24	26	26	106
		COP	20	30	14	5	69
		CP	11	11	25	4	51
		Uric acid	8	2	1	25	36
	%	CO	28.3	22.6	24.5	24.5	100.0
		COP	29.0	43.5	20.3	7.2	100.0
		CP	21.6	21.6	49.0	7.8	100.0
		Uric acid	22.2	5.6	2.8	69.4	100.0

Cross validation is done only for those cases in the analysis. In cross validation, each case is classified by the functions derived from all cases other than that case, resulting in 42.0% of original grouped cases correctly classified.

CO: calcium oxalate stone; COP: calcium oxalate phosphate; CP: calcium phosphate; UA: uric acid.

## Discussion

To date, no study showed specific predictive rate of stone composition using anthropometric parameters. Prevention strategy is only possible when the most affecting anthropometric parameters are established. Although many studies have shown the metabolic link between obesity and stone components, no study has determined the real role of anthropometric parameters in predicting calcium oxalate or uric acid stones associated with obesity. The most common reason for scanty is that direct measurement of visceral fat component and scientific analysis using specific statistical technique are difficult. Our study was the first study overcoming these two difficulties.

Obesity, an important public health problem in most countries, is associated with chronic medical conditions such as hypertension, hyperlipidemia, hypercholesterolemia, diabetes mellitus, cardiovascular disease, and other medical problems. The prevalence of urinary stone disease is higher in overweight or obese individuals. Recent studies have revealed that obesity is associated with changes in chemical components of serum and urine, such as citrate, phosphate, oxalate, and uric acid [5]. Metabolic change might explain the association between obesity and urinary stone disease. Higher BMI has been reported to be related with UA stones and CO stones [4,11-14]. Main explanations for such finding is that diabetes mellitus is correlated with insulin resistance or hyperinsulinemia, hyperuricosuria, salting-out effect in hyperuricosuria, and urine acidosis in obesity [4,11-13].

Before determining the relationship between obesity and stone composition, the relationship between obesity and urine acidosis has to be clarified. Urine pH was known to be negatively related with obesity which was verified in Asian patients with urolithiasis [15]. Our results showed a significant relationship among urinary pH, BMI, visceral fat, and total fat by correlation analysis. However, urinary pH was not related with stone composition in ANOVA test or discriminant analysis. Main reason for such result might be due to selection bias. Urinary pH had a diurnal variation. Urine collected in the morning had a significantly lower pH than urine collected in the day or evening [16]. We used a single urine sample collected at variable times, which could have distorted the results.

Although causal relationship between obesity and hyperuricosuria or uric acidosis have been reported in many studies [4,11-15], the role of obesity in determining stone component is still unclear without well documentation. Recent study has reported that the relationship between obesity and stone component was not so strong than their relationship reported before [6]. The main pitfalls of recent studies on obesity and stone composition are that they failed to include severe obese cohort with less application of visceral fat. One recent study answered one of these pitfalls [6]. Obesity has little effect on stone composition until a very high (>40) BMI is reached. The overall effect of metabolic syndrome on stone type is relatively small, because most stones are calcium oxalate and metabolic syndrome factors that have little impact on calcium oxalate frequency [6]. Metabolic syndrome has been linked to urinary stones, mainly uric acid stones [12,14]. Most studies about obesity and stone component dealt with BMI which is easy to calculate. However, BMI has fallen out of favor. There is a tendency that waist circumference or waist/hip ratio could replace it. Our previous report showed the usefulness of CT delineated measurement of visceral fat in showing its association with specific stone component [8].

To investigate whether morbidly obese patients have different physiology with respect to urine and stone composition compared to patients who are simply obese, measurement of visceral adiposity is essential. In our study, to measure visceral adiposity together with subcutaneous fat, waist circumference, and V/T ratio, we used CT delineation. CT delineation method, a validated method to measure visceral fat, has been used frequently [7].

One of the features of statistical analysis used in our study was that we used discriminant analysis, one validated multivariate analysis method used to adjust other confounding factors. By this analysis, we could overcome the retrospective nature of this study which might make selection bias. Moreover, we could find the most reliable factor to predict stone component.

Table 3 provides another way of indicating the relative importance of the predictors. Many researchers use this structure matrix correlations due to its superior accuracy than the Standardized Canonical Discriminant Function Coefficients. The structure matrix table (Table 3) shows the correlations among each variable with each discriminate function. These Pearson coefficients are structure coefficients or discriminant loadings. They serve like factor loadings in factor analysis. By identifying the largest loadings for each discriminate function the researcher gains insight into how to name each function. Here we have visceral fat, outer circumference and total fat which suggest effective factors as the function that discriminates between non-stone and stone. Generally, just like factor loadings, 0.40 is seen as the cut-off between important and less important variables.

This discriminant analysis involves the determination of a linear equation like regression anlaysis. The form of the equation or function is: D = v1 X1 + v2 X2 + v3 X3 + ...... +vi Xi + a, where D = discriminate function, v = the discriminant coefficient or weight for that variable, X = respondent’s score for that variable, a = constant, i = the number of predictor variables. This function is similar to a regression equation or function. The v’s are unstandardized discriminant coefficients analogous to the b’s in the regression equation. These v’s maximize the distance between the means of the criterion (dependent) variable. Standardized discriminant coefficients can also be used like beta weight in regression. Good predictors tend to have large weights. Discriminant analysis has merit with maximizing the distance between the categories, resulting in suggestion of an equation that has strong discriminatory power between groups. After using an existing set of data to calculate the discriminant function and classify cases, any new cases can then be classified. It operates just like a regression equation. In our case, discriminant function could be calculated using canonical discriminant function coefficients as shown in the following: discriminant function (D) = D = −5.767 + (0.733× sex) + (−0.023× age) + (−0.069× BMI) + (−0.030× visceral fat) + (0.011× subcutanous fat) + (0.426× viceral to subcutaneous fat ratio) + (0.062× visceral fat percentage) + (0.057× outer circumference) + (0.278× urine pH). By this equation, prediction was measured as 42.0%, indicating correct classification rate of the original group. By suture matrix, visceral fat was the largest absolute correlation between each variable and any discriminant function.

Although we have done a well designed retrospective pilot study, there are some limitations in this study. First, our sample population included only patients who underwent surgical intervention, which could not reflect the vast majority of patients with urinary stone disease. Second, we did not divided male and female which might have affected the results, because male and females might have difference in visceral fat adiposity and stone compositions. Obesity is known to be associated with the risk of urolithiasis not only in male adults but also in children and female patients [17]. To overcome this phenomenon, we used a discriminant analysis as mentioned above. Third, due to the retrospective nature of the study and the variation in practice among the clinicians at our institution, 24-hour urine data were unavailable or unobtainable. Fourth, the International Association for the Study of Obesity proposed the criterion for obesity to be BMI above 30 kg/m2, which was based on Caucasian data [18]. For people from Asia-Oceania who have the main energy intake from carbohydrates, obesity is defined as a BMI above 25 kg/m^2^. Finally, the role of diabetes mellitus, which might have some role in the formation of stone component, was not discussed in this study.

## Conclusions

Visceral fat measured by CT-based fat delineation was significantly associated with uric acid stone, suggesting that visceral fat has more important role in uric acid stone formation than total fat. Considering the effect of metabolic syndrome, visceral adiposity should be considered first instead of only BMI. Future studies on the preventing role of reducing visceral fat in urinary stone disease are merited.

## Abbreviations

BMI: Body mass index
CT: Computed tomography
V/T: Visceral fat to total fat ratio
CO: Calcium oxalate
COP: Calcium oxalate phosphate
CP: Calcium phosphate
UA: Uric acid
